# First Report of *Babesia microti*-Caused Babesiosis in Spain

**DOI:** 10.1089/vbz.2016.1946

**Published:** 2016-10-01

**Authors:** Marta Arsuaga, Luis M. Gonzalez, Cheryl A. Lobo, Fernando de la Calle, Jose M. Bautista, Isabel G. Azcárate, Sabino Puente, Estrella Montero

**Affiliations:** ^1^ Medicina Interna, Sección de Infecciosas and Unidad de Medicina Tropical y del Viajero, Hospital Carlos III, Madrid, Spain.; ^2^ Servicio de Parasitología, Centro Nacional de Microbiología, Majadahonda, Instituto de Salud Carlos III, Madrid, Spain.; ^3^ Blood-Borne Parasites Department, Lindsley Kimball Research Institute New York Blood Center, New York, New York.; ^4^ Medicina Interna, Sección de Infecciosas and Unidad de Medicina Tropical y del Viajero, Hospital Universitario La Paz, Madrid, Spain.; ^5^ Departamento de Bioquímica y Biología Molecular IV, Facultad de Veterinaria, Universidad Complutense, Madrid, Spain

**Keywords:** *Babesia*, diagnosis, zoonosis

## Abstract

Babesiosis is an emerging zoonosis now found in several areas of the world. Using PCR and indirect immunofluorescence assay, we have diagnosed the first case of human babesiosis caused by *Babesia microti* in Spain. Diagnosis was delayed because of the nonspecific clinical symptoms that occurred in an immunocompetent patient.

## Introduction

Human babesiosis is a zoonosis caused by intraerythrocytic protozoan parasites of the genus *Babesia*. Babesiosis is transmitted through the bite of infected *Ixodes* ticks, by blood transfusion or congenitally. Although fever is a hallmark of babesiosis, there are some nonspecific symptoms. This nonspecificity explains why a diagnosis of babesiosis might be delayed, or missed altogether (Vannier et al. 2015). *Babesia divergens* is considered the main agent of human babesiosis in Europe. However, cases of *Babesia microti*, *Babesia venatorum*, and *B. divergens*-like infections have also been described (Gray et al. 2010, Moniuszko-Malinowska et al. 2016).

## Case Study

A 35-year-old man with symptoms of fever, fatigue, and general malaise, but with normal splenic function, was admitted to a tertiary hospital in Spain in 2014. He had lived in Spain since 2003 and had visited Germany in 2012. The patient had traveled to Uruguay in 2013, a few days before the onset of symptoms. During a 10-day hospital stay, the patient had three episodes of fever and underwent an extensive diagnostic evaluation. All tests conducted were negative and the patient was discharged without a specific diagnosis. Four months later, the patient was admitted to the Unit for Tropical Diseases of the Hospital Carlos III in Madrid, Spain. He had persistent nonspecific symptoms such as the recurrence of fever, chills, headaches, weakness, general malaise, and constant fatigue over several months. As fever indicated an infectious disease, laboratory tests were carried out for a large number of pathogens, including *Anaplasma phagocytophilum*, *B. divergens*, and *B. microti*.

The results of Giemsa staining of thin blood smears (280–300 oil-immersion fields per slide) and conventional PCR tests specific for *B. divergens* (Gonzalez et al. 2014) and *B. microti* (Persing et al. 1992) were negative. DNA from *B. microti* Gray (ATCC^®^ 30221™) and *B. divergens* Bd Rouen 1987 strains was used as positive controls. The results of tests for *A*. *phagocytophilum* were also negative. Following the protocol for the diagnosis of babesiosis caused by *B. microti* (Vannier et al. 2015), tests were repeated a few days later. At this point, the patient had fever higher than 38°C. Although thin smear results remained negative, the *B. microti*-specific PCR test was positive. Using Bab1 and Bab4 primers (Persing et al. 1992), a 238-bp partial fragment of the *B. microti* 18S gene was amplified from the patient's blood, indicating parasite DNA positivity. The sequence of the amplicon (KT271759) showed 100% identity with the *B. microti* Munich strain 18S gene (AB366158.1 and AB071177). The amplicon also shared 100% identity with the *B. microti* 18S gene (AY789075.1) isolated from *Ixodes ricinus* ticks in Poland (Pieniazek et al. 2006). PCR tests for *B. divergens* remained negative.

Further confirmation was sought by injecting the patient's blood into a 6-week-old BALB/c mouse. We did not detect the parasite in the peripheral blood of the mouse. Nevertheless, the small number of parasites injected may have been sequestered within the spleen (Ruebush and Hanson 1979), because it was possible to amplify a 154-bp fragment from this organ using nested PCR (Persing et al. 1992) ∼4 weeks later. This amplicon had a 100% overlap with the PCR product obtained using the patient's blood. This result confirmed the presence of *B. microti*. To facilitate phylogenetic analysis, we attempted to amplify larger fragments of the *B. microti* 18S ribosomal and the β-tubulin genes from the patient's blood and the murine spleen, but this was not successful. A commercial indirect immunofluorescence assay (IFA) for *B. microti* was also performed (Fuller Laboratories). Using patient serum IgG, IFA titers of 1:64 were obtained.

The patient was immediately started on 750 mg oral atovaquone every 12 h and 500 mg oral azithromycin every 24 h for 7 days. After treatment, the patient still had a fever. Therefore, treatment with 250/100 mg atovaquone/proguanil and 500 mg azithromycin was initiated and carried out for 10 weeks without side effects. Although the patient still had frequent episodes of fever, his general condition improved. At the end of the treatment, the bouts of fever were resolved and the patient's only symptom was fatigue. In follow-up visits during the subsequent 4 months, PCR analysis revealed that the patient had cleared the parasites.

## Discussion

In Europe, human babesiosis is not notifiable continent wide. Therefore, not all clinics are fully cognizant of the disease and it is likely that cases are underreported.

To our knowledge, we report the first known PCR-confirmed case of *B. microti*-babesiosis in Spain. However, this case was also marked by the absence of parasites in four blood smears acquired at two different times, low antibody levels, and a poor response to specific therapy.

The source of infection was not determined. The patient owned a dog but did not recall being exposed to ticks. He had visited and lived in European countries where *B. microti* had been detected (Estrada-Pena et al. 2005, Obiegala et al. 2015). However, his symptoms began only after traveling to Uruguay, where there is no clear information regarding the feeding habits of *Ixodes* ticks (Guglielmone et al. 2006).

Of note, there are many similarities between the clinical profiles of this patient and the six recently diagnosed babesiosis cases in Poland (Moniuszko-Malinowska et al. 2016). All the patients had nonspecific symptoms. *B. microti* parasites were not detected through Giemsa smears, probably because the parasitemia was lower than 0.1% (babesiosis typically presents at parasitemias >0.1%) (Teal et al. 2012). The serology was positive by IFA (1:64) in only some of the patients, but the molecular diagnostics confirmed the presence of *B. microti* in all cases. The partial *B. microti* 18S gene sequences from both studies were identical to those of the Munich strain (AB071177), which has so far not been implicated in human disease (Sinski et al. 2006) and the strain from Eurasia (AY789075.1), already mentioned. Notably, the Munich and the Eurasia strains infect *I. ricinus* (Pieniazek et al. 2006, Sinski et al. 2006). Both form a cluster that is independent of the cluster to which the European Jena and North American *B. microti* strains, isolated from humans, belong (Pieniazek et al. 2006).

The quantity and quality of the DNA samples, which we obtained, probably limited an in-depth characterization of the causative *B. microti* strain. Thus, large fragments of 18S rDNA from *B. microti* from human infections are still needed to discriminate precisely the strain involved.

Although the patients from Poland recovered without therapy, the patient we report responded poorly to antibabesial drugs. Thus, attention should be paid to the treatment of patients with nonspecific symptoms.

## Conclusions

This study describes an example of low-grade chronic human babesiosis caused by *B. microti*, which may affect many immunocompetent patients. Diagnosis of these cases can be truly challenging in the face of negative blood smears and nonspecific symptoms. Advances in diagnosis and treatment require standardized microscopy protocols, based on the frequency and number of slides per patient, as well as more sensitive molecular protocols, which could be used to identify the specific disease-causing strain of the parasite. Results from such testing could then be used to guide the use of specific therapy. Moreover, the dynamics of transmission of European *B. microti* strains must be examined in depth to prevent risks to human health, to minimize blood supply contamination, and to overcome the challenge of reliably diagnosing this growing worldwide zoonosis.

## Ethics Statement

The animal procedure and the protocol were approved by the Consejería de Medio Ambiente, Administración Local y Ordenación del Territorio de la Comunidad de Madrid, Spain.
